# Temporal Trends and Demographic Disparities in Abdominal Aortic Aneurysm Mortality Among U.S. Adults Aged ≥ 65 Years, 1999–2024: A Nationwide Population-Based Analysis of CDC WONDER Data

**DOI:** 10.3390/jcm15135130

**Published:** 2026-07-01

**Authors:** Hassaan Abid, Muhammad Jawad, Sohana Memon, Muhammad Vazaym, Gaaitri Lohano, Rimsha Adnan, Muhammad Mohid Haroon, Rithik Khiani

**Affiliations:** 1 Department of Internal Medicine, Indiana University School of Medicine, Indianapolis, IN 46202, USA; 2 Department of Internal Medicine, Liaquat University of Medical and Health Sciences, Jamshoro 76090, Pakistan; 3 Department of Internal Medicine, Dow University of Health Sciences (DUHS), Karachi 75600, Pakistan; 4 Department of Internal Medicine, Ameer-Ud-Din Medical College, Lahore 54000, Pakistan; 5 Department of Internal Medicine, Jinnah Sindh Medical University, Karachi 75500, Pakistan

**Keywords:** abdominal aortic aneurysm, mortality trends, CDC WONDER, health disparities, joinpoint analysis, vascular epidemiology

## Abstract

**Background:** Abdominal aortic aneurysm (AAA) remains an important cause of cardiovascular mortality in the United States despite advances in screening, surveillance, and aneurysm repair. Contemporary national analyses evaluating long-term AAA mortality trends across demographic and geographic subgroups remain limited, particularly following the COVID-19 pandemic period. **Objective:** To evaluate temporal trends in AAA-related mortality among U.S. adults aged ≥ 65 years from 1999 to 2024 and characterize demographic and geographic disparities. **Methods:** A retrospective population-based study was conducted using the CDC WONDER Multiple Cause of Death database. AAA-related deaths were identified using International Classification of Diseases, Tenth Revision (ICD-10) codes I71.3 and I71.4. Age-adjusted mortality rates (AAMRs) per 100,000 population were calculated using the 2000 U.S. standard population. Mortality trends were stratified by sex, age, race/ethnicity, census region, and urbanization status. Joinpoint regression analysis was used to estimate annual percent change (APC) and average annual percent change (AAPC). **Results:** Between 1999 and 2024, a total of 208,476 AAA-related deaths among U.S. adults aged ≥ 65 years were recorded. Overall AAMR declined significantly from 32.61 per 100,000 population in 1999 to 12.17 per 100,000 population in 2024 (AAPC −3.86%; 95% confidence interval [CI]: −4.43 to −3.30; *p* < 0.001). Mortality rates remained consistently higher among males, adults aged ≥ 85 years, non-Hispanic White individuals, residents of the Midwest, and non-metropolitan populations. Joinpoint analysis demonstrated sustained declines across most demographic groups. Although several subgroups exhibited a temporary plateau in declining mortality trends during the pandemic era, these trend changes did not reach statistical significance. Following 2021, mortality rates resumed a significant downward trend. **Conclusions:** AAA-related mortality among U.S. adults aged ≥ 65 years declined substantially between 1999 and 2024; however, important demographic and geographic disparities persist. Continued efforts aimed at risk factor reduction, equitable screening access, and preventive vascular care remain essential to further reduce AAA-related mortality among older adults.

## 1. Introduction

Abdominal aortic aneurysm (AAA) is a progressive dilation of the abdominal aorta and remains an important cause of cardiovascular morbidity and mortality worldwide [1]. Ruptured AAA is associated with extremely high mortality, with many patients dying before reaching medical care and perioperative mortality remaining substantial even among those undergoing emergent repair [2,3]. Given these poor outcomes, early detection and elective management remain central to reducing AAA-related mortality.

AAA development is strongly associated with advanced age, male sex, smoking, hypertension, and atherosclerotic cardiovascular disease [4,5,6]. Although AAA is more prevalent among men, important sex-related differences in disease presentation and outcomes have been reported. Women with AAA may experience delayed diagnosis, rupture at smaller aneurysm diameters, less favorable anatomic characteristics for endovascular repair, and higher perioperative morbidity and mortality compared with men. These observations underscore the importance of evaluating AAA mortality trends separately among males and females and considering sex-specific factors when interpreting population-level outcomes [7,8].

Over the past two decades, mortality related to AAA has declined significantly in several developed countries, including the United States [9,10,11]. This reduction has likely been driven by improvements in cardiovascular risk factor modification, declining smoking prevalence, implementation of screening programs, and advances in surgical and endovascular aneurysm repair techniques [12,13,14,15,16,17,18,19,20,21]. Despite these advances, important demographic and geographic disparities in AAA burden continue to persist across the United States [10,11].

The COVID-19 pandemic created major disruptions in healthcare delivery systems worldwide, including reductions in outpatient care, delayed diagnostic evaluation, and postponement of elective surgical procedures [22,23,24,25,26,27,28,29,30,31]. These disruptions raised concerns regarding the indirect effects of the pandemic on the diagnosis and management of chronic vascular diseases such as AAA.

Previous studies have demonstrated declining AAA mortality trends in the United States; however, contemporary analyses incorporating post-pandemic national data and detailed subgroup characterization remain limited [10,11]. Consequently, it remains uncertain whether the long-standing decline in AAA-related mortality persisted during and after the COVID-19 pandemic and whether demographic and geographic disparities have changed over time. An updated national analysis through 2024 is therefore warranted to evaluate contemporary mortality patterns and provide insight into the evolving burden of AAA-related mortality in the United States.

Therefore, this study aimed to evaluate temporal trends in AAA-related mortality in the United States from 1999 through 2024 using the CDC WONDER Multiple Cause of Death database. We further examined mortality patterns across sex, age, race/ethnicity, geographic region, and urbanization subgroups to better characterize persistent disparities in AAA-related mortality.

## 2. Methods

### 2.1. Study Design and Data Source

This retrospective population-based study utilized mortality data from the Centers for Disease Control and Prevention (CDC) Wide-Ranging Online Data for Epidemiologic Research (WONDER) Multiple Cause of Death (MCOD) database between 1999 and 2024 [32]. The MCOD database contains national death certificate data collected from all 50 U.S. states and the District of Columbia and includes both underlying and contributing causes of death.

AAA-related deaths were identified using International Classification of Diseases, Tenth Revision (ICD-10) codes I71.3 (ruptured abdominal aortic aneurysm) and I71.4 (abdominal aortic aneurysm without rupture) [10].

The Multiple Cause of Death (MCOD) database was used to identify deaths in which abdominal aortic aneurysm (AAA) was listed anywhere on the death certificate, regardless of whether it was recorded as the underlying or a contributing cause of death. This approach was selected to capture the broader burden of AAA-related mortality, whereas restricting analyses to the underlying cause of death alone may underestimate the overall contribution of AAA to mortality at the population level.

The study period was divided into three predefined intervals for descriptive temporal assessment: pre-pandemic (1999–2019), pandemic-era (2020–2021), and post-pandemic (2022–2024). Because the CDC WONDER database contains publicly available de-identified data, institutional review board approval and informed consent were not required. This study was conducted in accordance with the Strengthening the Reporting of Observational Studies in Epidemiology (STROBE) reporting recommendations for observational studies [33].

### 2.2. Study Variables

Extracted variables included year of death, age, sex, race/ethnicity, census region, urbanization status, and place of death. Age-stratified analyses were limited to adults aged ≥ 65 years and categorized as 65–74 years, 75–84 years, and ≥85 years because AAA-related mortality predominantly occurs among older adults, whereas mortality counts among younger individuals are comparatively low.

Race and ethnicity were classified according to CDC WONDER definitions as non-Hispanic White, non-Hispanic Black or African American, non-Hispanic American Indian or Alaska Native, non-Hispanic Asian or Pacific Islander, and Hispanic or Latino. Geographic analyses were performed using U.S. Census regions, including the Northeast, Midwest, South, and West.

Urbanization status was categorized according to the National Center for Health Statistics urban-rural classification scheme and grouped into metropolitan and non-metropolitan areas. Urbanization analyses were restricted to 1999–2020 because county-level urbanization classifications were not available in the CDC WONDER dataset for subsequent years at the time of analysis.

### 2.3. Statistical Analysis

Crude mortality rates and age-adjusted mortality rates (AAMRs) per 100,000 population were calculated using CDC WONDER standard methodology. Age adjustment was performed using the 2000 U.S. standard population.

Temporal mortality trends were evaluated using Joinpoint regression analysis [34]. Annual percent change (APC) and average annual percent change (AAPC) with corresponding 95% confidence intervals (CIs) were calculated to assess changes in mortality trends over time. The Monte Carlo permutation method was used to identify the optimal number of joinpoints. A maximum of four joinpoints was specified to balance model flexibility with the risk of overfitting. Joinpoint regression models were fitted using the standard error estimates provided for each annual mortality rate. Heteroscedastic errors were accounted for through the standard error-based modeling approach implemented in the Joinpoint Regression Program, and an uncorrelated error structure was specified. The final model selection was based on permutation testing, which determined the best-fitting model for the observed temporal trends.

No CDC WONDER mortality counts were suppressed in the extracted dataset. Accordingly, all available subgroup estimates were included in the analyses.

Trend analyses were stratified according to sex, age group, race/ethnicity, census region, and urbanization status. Statistical significance was defined as a two-sided *p*-value < 0.05. All analyses were performed using the Joinpoint Regression Program, version 5.4 [34].

A sensitivity analysis was also conducted by restricting ICD-10 codes (I71.3 and I71.4) to the Underlying Cause of Death (UCOD) dataset. This approach assessed whether limiting the definition to the underlying cause of death would meaningfully alter the mortality patterns observed in the primary analysis. Additionally, sensitivity analyses were performed by separately evaluating I71.3 and I71.4 within the Multiple Cause of Death (MCOD) dataset to determine whether trends differed when ruptured and non-ruptured abdominal aortic aneurysms were analyzed independently. These complementary analyses were undertaken to ensure the consistency and robustness of the findings across different coding and classification frameworks within CDC WONDER.

## 3. Results

### 3.1. Overall

Between 1999 and 2024, a total of 208,476 AAA-related deaths among adults aged ≥ 65 were recorded in the United States. The overall age-adjusted mortality rate (AAMR) declined substantially from 32.61 per 100,000 population in 1999 to 12.17 per 100,000 population in 2024, corresponding to a significant overall decrease throughout the study period (AAPC −3.86%; 95% confidence interval [CI]: −4.43 to −3.30; *p* < 0.001).

**Figure 1:**
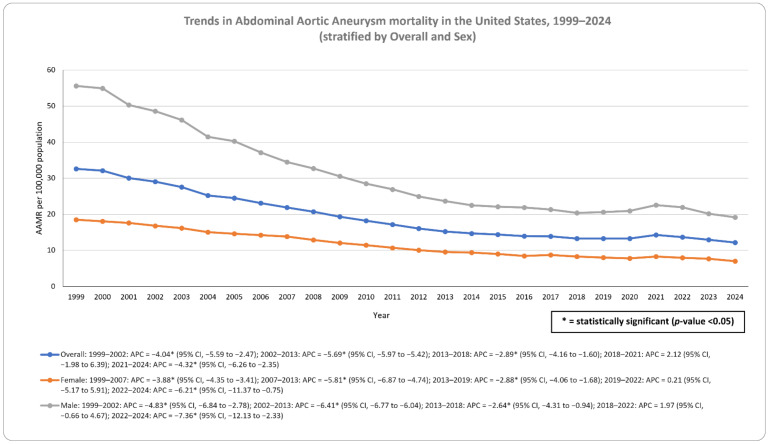
Overall and Sex-Stratified Age-adjusted mortality rates (per 100,000) for Abdominal Aortic Aneurysm mortality among U.S. adults aged ≥ 65 years, 1999–2024.

**Figure 2:**
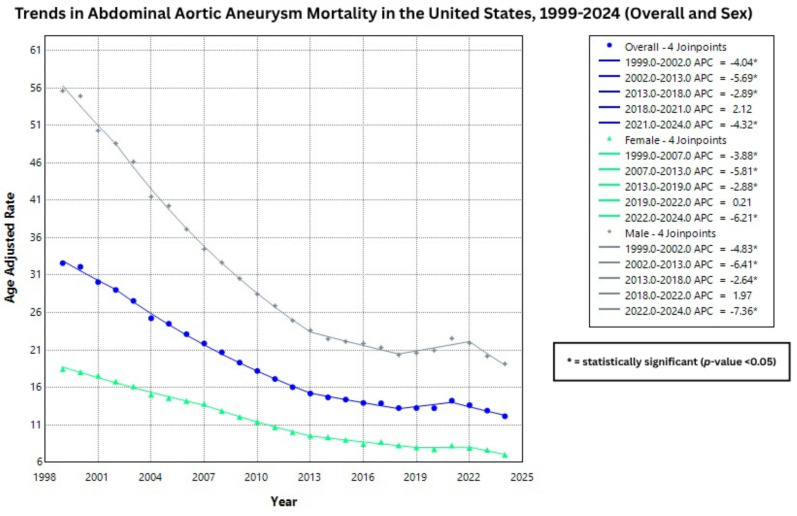
Joinpoint Analysis of Overall and Sex-Stratified Age-adjusted mortality rates (per 100,000) for Abdominal Aortic Aneurysm mortality among U.S. adults aged ≥ 65 years, 1999–2024.

Joinpoint analysis identified multiple temporal phases in mortality trends. Mortality decreased significantly between 1999 and 2002 (APC −4.04%; 95% CI: −5.59 to −2.47; *p* < 0.001), followed by a steeper decline from 2002 to 2013 (APC −5.69%; 95% CI: −5.97 to −5.42; *p* < 0.001). A continued but slower decline was observed from 2013 to 2018 (APC −2.89%; 95% CI: −4.16 to −1.60; *p* < 0.001). Between 2018 and 2021, mortality trends demonstrated temporary plateauing without statistical significance (APC 2.12%; 95% CI: −1.98 to 6.39; *p* = 0.287), followed by resumption of a significant downward trend from 2021 to 2024 (APC −4.32%; 95% CI: −6.26 to −2.35; *p* < 0.001) (Figure 1 and Figure 2) (Supplementary Tables S1 and S2).

### 3.2. Sex Stratified

AAA-related mortality remained consistently higher among males throughout the study period. Mean AAMR was 31.14 per 100,000 population among males compared with 11.61 per 100,000 population among females. Among males, mortality declined from 55.61 per 100,000 population in 1999 to 19.17 per 100,000 population in 2024, whereas among females mortality decreased from 18.48 to 7.02 per 100,000 population during the same interval.

Females demonstrated a significant overall decline in mortality across the study period (AAPC −3.82%; 95% CI: −4.60 to −3.03; *p* < 0.001). Mortality declined significantly from 1999 to 2007 and from 2007 to 2013, followed by a more gradual decline between 2013 and 2019. Mortality trends remained stable from 2019 to 2022 before declining significantly again between 2022 and 2024.

Similarly, males demonstrated a significant overall reduction in mortality (AAPC −4.25%; 95% CI: −4.90 to −3.60; *p* < 0.001). Significant decreases were observed between 1999 and 2018, followed by a temporary non-significant plateau between 2018 and 2022 and a renewed significant decline after 2022 (Figure 1 and Figure 2) (Supplementary Tables S1 and S2).

### 3.3. Race Stratified

Mortality patterns varied substantially across racial and ethnic groups. Non-Hispanic White individuals demonstrated the highest mean AAMR (21.16 per 100,000 population), followed by American Indian or Alaska Native individuals (15.28), non-Hispanic Black individuals (12.64), Asian or Pacific Islander individuals (10.79), and Hispanic individuals (8.77).

**Figure 3:**
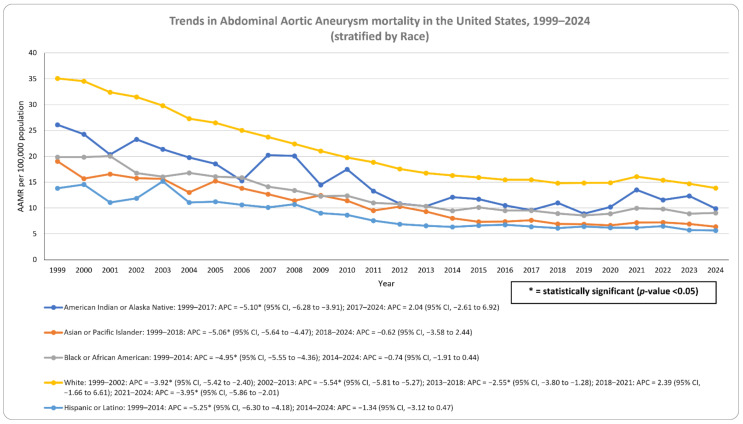
Race-stratified Age-adjusted mortality rates (per 100,000) for Abdominal Aortic Aneurysm mortality among U.S. adults aged ≥ 65 years, 1999–2024.

All racial and ethnic groups demonstrated overall declining mortality trends during the study period. Hispanic individuals experienced a significant mortality decline from 1999 to 2014, followed by relative stabilization thereafter. American Indian or Alaska Native populations demonstrated declining mortality through 2017, followed by a non-significant upward trend. Asian or Pacific Islander individuals experienced sustained mortality reductions through 2018, with stable trends subsequently observed. Non-Hispanic Black individuals demonstrated significant mortality declines until 2014, after which mortality trends plateaued. Non-Hispanic White individuals demonstrated sustained reductions in mortality throughout most of the study period, although temporary non-significant plateauing was observed between 2018 and 2021 before mortality resumed declining after 2021 (Figure 3) (Supplementary Tables S1 and S2).

### 3.4. Age Stratified

**Figure 4:**
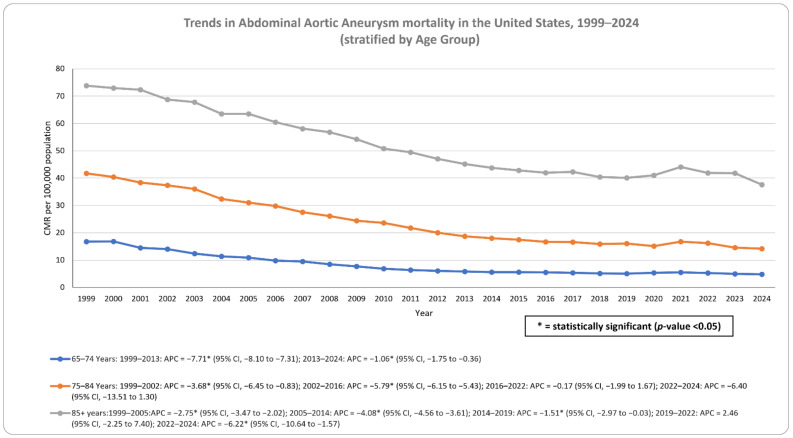
Age group-Stratified Crude-mortality rates (per 100,000) for Abdominal Aortic Aneurysm mortality among U.S. adults aged ≥ 65 years, 1999–2024.

AAA-related mortality increased substantially with advancing age. Mean crude mortality rates were highest among adults aged ≥ 85 years (52.42 per 100,000 population), followed by individuals aged 75–84 years (24.12) and 65–74 years (8.31). Individuals aged 65–74 years demonstrated the largest overall mortality reduction during the study period (AAPC −4.84%; 95% CI: −5.19 to −4.48; *p* < 0.001), with steep declines observed between 1999 and 2013 followed by slower continued reductions thereafter. Adults aged 75–84 years also demonstrated significant overall mortality reductions (AAPC −4.27%; 95% CI: −5.03 to −3.50; *p* < 0.001), although mortality trends stabilized between 2016 and 2022. Among adults aged ≥ 85 years, mortality declined more gradually overall (AAPC −2.66%; 95% CI: −3.35 to −1.97; *p* < 0.001), with temporary non-significant increases observed during the pandemic era before mortality resumed declining after 2022 (Figure 4) (Supplementary Tables S1 and S2).

### 3.5. Census Region

Geographic variation in AAA-related mortality was observed across U.S. census regions. The Midwest demonstrated the highest mean AAMR (22.43 per 100,000 population), followed by the Northeast (19.36), West (18.46), and South (17.80).

**Figure 5:**
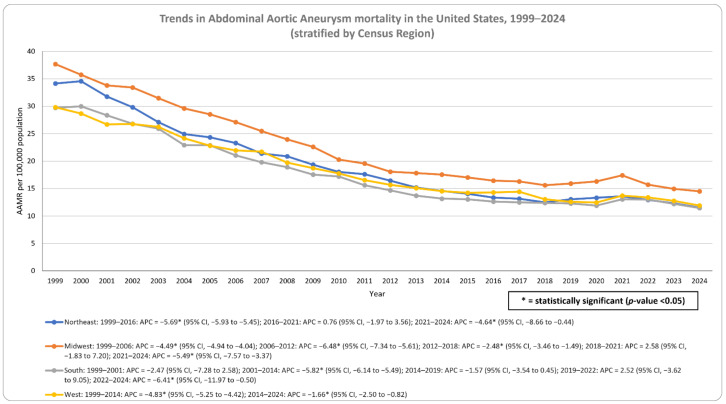
Census Region-Stratified Age-adjusted mortality rates (per 100,000) for Abdominal Aortic Aneurysm mortality among U.S. adults aged ≥ 65 years, 1999–2024.

All census regions demonstrated significant long-term declines in mortality. The Northeast exhibited the largest overall reduction (AAPC −4.31%; 95% CI: −5.00 to −3.62; *p* < 0.001). The Midwest and South demonstrated temporary non-significant plateauing during the pandemic era before significant mortality declines resumed after 2021–2022. Mortality in the West declined more steadily throughout the study period without substantial interruption (Figure 5) (Supplementary Tables S1 and S2).

### 3.6. Urbanization

AAA-related mortality differed according to urbanization status. Metropolitan areas accounted for 140,609 deaths, whereas non-metropolitan areas accounted for 39,428 deaths. Mean AAMR remained higher in non-metropolitan regions (24.48 per 100,000 population) compared with metropolitan areas (19.54).

**Figure 6:**
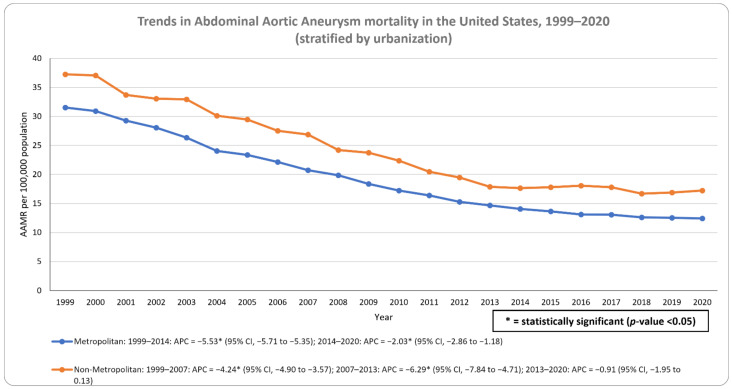
Urbanization-Stratified Age-adjusted mortality rates (per 100,000) for Abdominal Aortic Aneurysm mortality among U.S. adults aged ≥ 65 years, 1999–2020.

Both metropolitan and non-metropolitan areas demonstrated significant overall mortality reductions. Metropolitan regions experienced a sustained decline throughout the study period (AAPC −4.54%; 95% CI: −4.79 to −4.29; *p* < 0.001). Non-metropolitan regions demonstrated significant mortality reductions through 2013, followed by relative stabilization thereafter (AAPC −3.74%; 95% CI: −4.31 to −3.17; *p* < 0.001) (Figure 6) (Supplementary Tables S1 and S2).

### 3.7. State

**Figure 7:**
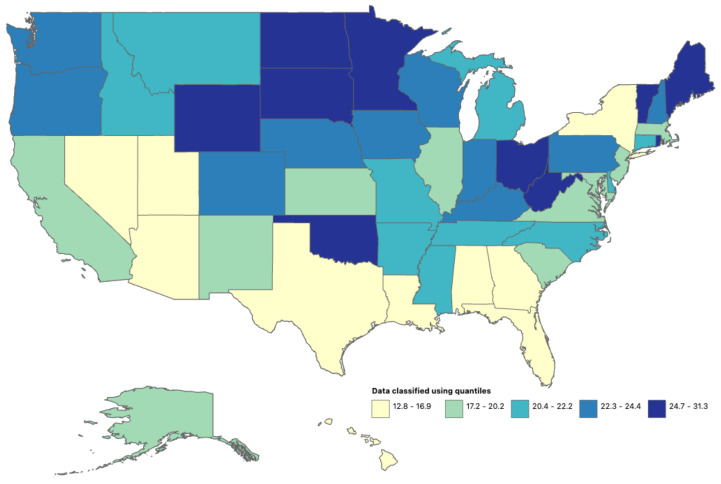
State-Stratified Age-adjusted mortality rates (per 100,000) for Abdominal Aortic Aneurysm mortality among U.S. adults aged ≥ 65 years, 1999–2024.

Substantial geographic variation in mortality rates was observed at the state level. West Virginia demonstrated the highest AAMR (29.32 per 100,000 population), followed by Vermont, Wyoming, Minnesota, Maine, and South Dakota. In contrast, lower mortality rates were observed in Utah, the District of Columbia, Georgia, Arizona, Texas, and Florida (Figure 7).

### 3.8. Place of Death

**Figure 8:**
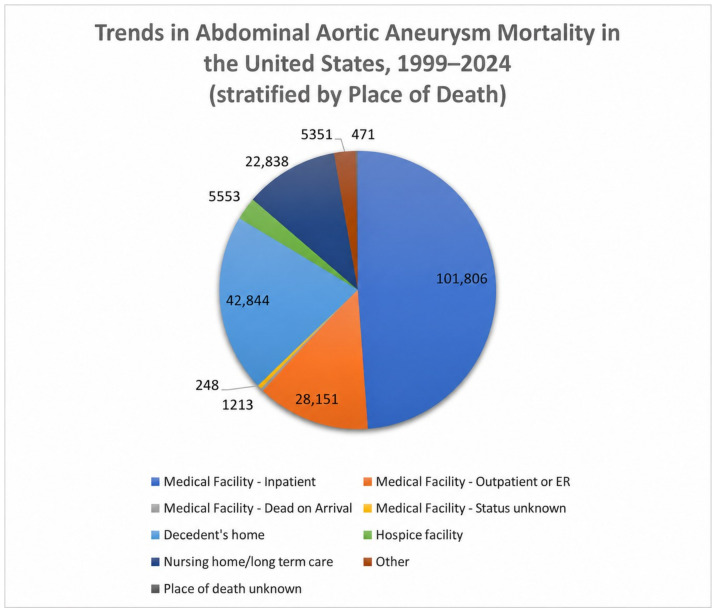
Place of Death stratified Abdominal Aortic Aneurysm mortality among U.S. adults aged ≥ 65 years, 1999–2024.

Most AAA-related deaths occurred in inpatient medical facilities (101,806 deaths), followed by decedents’ residences (42,844 deaths) and outpatient or emergency department settings (28,151 deaths). Additional deaths occurred in nursing facilities or long-term care centers (22,838 deaths), hospice facilities (5553 deaths), and other reported locations (Figure 8).

### 3.9. Sensitivity Analysis

To assess the robustness of the primary findings, a sensitivity analysis was performed restricting deaths to those in which abdominal aortic aneurysm (AAA) was recorded as the underlying cause of death. AAMRs decreased from 20.97 per 100,000 population in 1999 to 4.94 per 100,000 population in 2024 (AAPC: −5.74%; 95% CI: −6.35 to −5.13; *p* < 0.000001). Mortality rates declined significantly from 1999 to 2006 (APC: −5.40%; 95% CI: −6.34 to −4.46; *p* < 0.000001), followed by a steeper decline from 2006 to 2011 (APC: −9.46%; 95% CI: −11.99 to −6.86; *p* = 0.000001), and a continued but more gradual decrease from 2011 to 2024 (APC: −4.46%; 95% CI: −4.99 to −3.92; *p* < 0.000001) (Supplementary Figure S1). These findings were directionally consistent with the primary MCOD-based analysis, demonstrating a sustained decline in AAA-related mortality throughout the study period.

### 3.10. Ruptured AAA-Related Mortality

For ruptured abdominal aortic aneurysm (AAA), AAMRs decreased from 15.71 in 1999 to 3.32 in 2024 (AAPC: −6.07; 95% CI: −6.43 to −5.71, *p* < 0.000001). Mortality rates declined significantly from 1999 to 2014 (APC: −6.95; 95% CI: −7.28 to −6.62, *p* < 0.000001), followed by a continued significant decline from 2014 to 2024 (APC: −4.74; 95% CI: −5.56 to −3.91, *p* < 0.000001) (Supplementary Figure S2).

### 3.11. Non-Ruptured AAA-Related Mortality

For non-ruptured abdominal aortic aneurysm (AAA), AAMRs decreased from 16.96 in 1999 to 9.31 in 2024 (AAPC: −2.38; 95% CI: −3.07 to −1.68, *p* < 0.000001). Mortality rates remained stable from 1999 to 2001 (APC: −0.39; 95% CI: −4.33 to 3.71, *p* = 0.836), followed by a significant decline from 2001 to 2014 (APC: −4.27; 95% CI: −4.52 to −4.02, *p* < 0.000001). Mortality rates again remained stable from 2014 to 2018 (APC: −0.90; 95% CI: −3.24 to 1.49, *p* = 0.423), followed by a non-significant upward trend from 2018 to 2021 (APC: 3.97; 95% CI: −0.63 to 8.79, *p* = 0.085), and a significant decline from 2021 to 2024 (APC: −3.50; 95% CI: −5.58 to −1.38, *p* = 0.004) (Supplementary Figure S3).

## 4. Discussion

In this nationwide analysis of AAA-related mortality in the United States from 1999 through 2024, several important findings were observed. First, AAA mortality declined substantially over the study period, with significant reductions in age-adjusted mortality rates across nearly all demographic and geographic subgroups. Second, despite these overall improvements, marked disparities persisted according to sex, age, race/ethnicity, geographic region, and urbanization status. Third, although temporary plateauing in declining mortality trends was observed during the pandemic era, these changes did not reach statistical significance, and mortality rates resumed declining thereafter.

The sustained reduction in AAA-related mortality observed in this study is consistent with prior national and international epidemiologic analyses demonstrating declining AAA mortality over recent decades [10,11,12,13]. It is important to note that this study employed a multiple-cause-of-death methodology, including deaths in which AAA was listed as either an underlying or contributing cause. To address this methodological consideration, a sensitivity analysis restricted to deaths where AAA was the underlying cause of death was performed, which confirmed a similarly robust long-term decline in mortality, supporting the validity of the primary findings. Similarly, sensitivity analyses stratified by ruptured versus non-ruptured AAA demonstrated sustained and significant declines in both groups. Multiple factors may have plausibly contributed to this observed improvement, although the present database does not permit direct causal attribution. Declining smoking prevalence in the United States may have played a role, given the well-established association between tobacco exposure and AAA formation, growth, and rupture risk [14,15]. Improvements in cardiovascular risk factor management, including hypertension and lipid control, may have additionally contributed to reduced aneurysm progression and rupture risk [16,17]. Furthermore, implementation of screening programs among high-risk populations and advances in elective aneurysm repair may have improved early detection and management of AAA [18,19,20,21]. However, because the CDC WONDER database does not include patient-level data on smoking history, screening participation, aneurysm diameter, rupture status, or repair modality, these explanations should be regarded as plausible hypotheses rather than demonstrated causal mechanisms.

Consistent with prior literature, males demonstrated substantially higher mortality rates compared with females throughout the study period [7,35,36,37,38,39,40]. This disparity likely reflects the greater prevalence of AAA among males as well as differences in smoking exposure and vascular risk factor burden [38,39,40].

However, the relationship between sex and AAA outcomes is considerably more complex than prevalence differences alone. Although females demonstrated lower overall mortality rates in this analysis, women with AAA face several clinically important disadvantages that may contribute to worse outcomes once disease is present. Women have historically been underrepresented in or excluded from population-based AAA screening programs, which in many countries have been designed primarily for older male smokers, potentially resulting in delayed diagnosis in female patients [7]. Furthermore, women with AAA are known to experience rupture at smaller aortic diameters compared with men, and may have more hostile proximal aortic neck morphology, smaller access vessel caliber, and reduced eligibility for standard endovascular repair in certain anatomic configurations, all of which may translate to greater perioperative risk and fewer treatment options [7,8]. Recent systematic reviews and cohort studies have further documented persistent sex-specific disparities in outcomes following endovascular aortic aneurysm repair (EVAR), with female patients demonstrating higher rates of perioperative complications, reinterventions, and mortality compared with male patients undergoing similar procedures [8]. These observations suggest that the lower absolute AAA mortality observed among women should not be interpreted as evidence of equivalent outcomes. Rather, sex-specific differences in disease presentation, aneurysm anatomy, screening eligibility, and perioperative risk may contribute to persistent disparities in AAA-related outcomes reported in both the present study and prior literature. Future risk-based screening strategies that account for cardiovascular risk factors beyond sex alone, including smoking history and familial risk, may be warranted to achieve earlier diagnosis and more equitable outcomes in female patients. Importantly, mortality trends declined significantly among both sexes, suggesting that advances in prevention and treatment have conferred broad population-level benefit; however, whether these improvements have occurred proportionally and equitably between men and women requires further investigation.

Persistent racial and ethnic disparities in AAA mortality were also observed. Non-Hispanic White individuals demonstrated the highest mortality rates, consistent with prior epidemiologic studies identifying White race as a major risk factor for AAA development [10,41,42,43]. In contrast, Hispanic and Asian or Pacific Islander populations demonstrated lower mortality rates throughout the study period. These differences may partially reflect variation in baseline AAA prevalence and cardiovascular risk factor burden; however, disparities in healthcare access, screening utilization, and disease recognition may also contribute to these findings [42,43]. Although mortality declined across all racial and ethnic groups, several populations demonstrated recent trend stabilization, suggesting that reductions in AAA burden have not occurred uniformly.

Age-stratified analyses demonstrated progressively higher mortality rates with advancing age, within the study population aged ≥ 65 years, mortality increased progressively with age and was highest among adults aged ≥ 85 years. While mortality declined significantly across all age groups, the magnitude of improvement was smaller among older adults. These findings likely reflect the increasing prevalence of AAA with aging as well as greater competing comorbidity burden and reduced suitability for operative intervention among elderly populations [42,44].

Significant geographic and urban–rural variation in mortality was additionally observed, with the Midwest and non-metropolitan regions demonstrating consistently higher mortality rates compared with other regions and metropolitan areas. These findings may reflect a combination of regional factors, including higher smoking prevalence, greater burden of cardiovascular risk factors such as hypertension and dyslipidemia, socioeconomic deprivation, and differential access to vascular specialty care [45,46,47]. In rural and non-metropolitan settings, excess mortality may also be driven by limited availability of screening ultrasound programs, reduced proximity to centers offering elective open or endovascular aneurysm repair, longer emergency transport times for ruptured AAA, and lower hospital procedural volumes for complex vascular surgery. These structural limitations likely reduce opportunities for elective repair and increase the proportion of deaths occurring after rupture or emergency presentation. These disparities highlight the need to improve equitable access to AAA screening and vascular surgical services in underserved and rural populations.

A temporary plateau in declining mortality trends was observed during the pandemic-era interval across several demographic subgroups and geographic regions. This observation may be contextually consistent with broader disruptions in healthcare delivery during the COVID-19 pandemic, including delays in outpatient evaluation, reduced access to diagnostic imaging, postponement of elective procedures, and avoidance of healthcare utilization [25,26]. However, these trend changes did not reach statistical significance in the primary analysis. Accordingly, the observed plateau should be interpreted as a descriptive and hypothesis-generating finding rather than evidence of a direct pandemic-related effect on AAA mortality. Given the ecological design of the study and the absence of patient-level clinical data, causal inferences cannot be made regarding the impact of COVID-19 on AAA-related mortality outcomes. The observed distribution of place of death provides additional insight into AAA-related mortality patterns. Most deaths occurred within inpatient medical facilities, reflecting the severe nature of AAA-related presentations and the high mortality associated with aneurysm rupture [2,3]. A substantial proportion of deaths also occurred at home, which may reflect delayed recognition of symptoms, sudden aneurysm rupture, or limited access to emergent medical care.

## 5. Limitations

This study has several important limitations. First, the analysis relied on death certificate data, which are subject to coding inaccuracies, misclassification bias, and variability in ICD-10 coding practices. Although the multiple-cause-of-death approach is appropriate for assessing AAA-related mortality burden, deaths in which AAA was listed solely as a contributing condition may not represent true AAA-attributable mortality; however, sensitivity analyses restricted to underlying-cause deaths yielded consistent findings. Second, the CDC WONDER database lacks detailed clinical information, including aneurysm characteristics, smoking history, comorbidities, treatment modality, and operative urgency, precluding assessment of disease severity and treatment-related factors. Third, the absence of screening and surveillance data prevents evaluation of the contribution of AAA screening programs to observed mortality trends. Fourth, because this was an observational ecological analysis, causal relationships cannot be inferred. Fifth, the study could not adjust for regional differences in smoking prevalence, healthcare access, socioeconomic factors, or other population-level determinants, limiting interpretation of geographic comparisons. Sixth, subgroup estimates for smaller racial/ethnic groups or less populated states may be subject to statistical instability because of low event counts. Seventh, the relatively short post-pandemic follow-up period limits assessment of longer-term mortality patterns following the COVID-19 era. Eighth, urbanization-stratified analyses were limited to 1999–2020 because urbanization data were unavailable for 2021–2024. Finally, the study population was restricted to adults aged ≥ 65 years, limiting generalizability to younger populations.

## 6. Conclusions

Despite these limitations, this study provides a contemporary nationwide assessment of AAA-related mortality trends using a large population-based database spanning more than two decades. The findings demonstrate substantial long-term reductions in AAA mortality in the United States while highlighting persistent demographic and geographic disparities. Continued efforts focused on smoking cessation, equitable screening access, cardiovascular risk reduction, and timely vascular care remain important to further reduce AAA-related mortality nationwide.

## Data Availability

The data that support the findings of this study are publicly available from the Centers for Disease Control and Prevention (CDC) Wide-ranging Online Data for Epidemiologic Research (CDC WONDER) Multiple Cause of Death database (https://wonder.cdc.gov/). These data are de-identified and accessible without restriction. Because the dataset is publicly available and contains no identifiable personal information, institutional review board approval and informed consent were not required.

## Supplementary Materials

The following supporting information can be downloaded at: https://www.mdpi.com/article/10.3390/jcm15135130/s1, Figure S1: Sensitivity Analysis; Figure S2: Trends in Abdominal Aortic Aneurysm (ruptured) Mortality in the United States, 1999–2024; Figure S3: Trends in Abdominal Aortic Aneurysm (Non-ruptured) Mortality in the United States, 1999–2024; Table S1: Demographic Characteristics, Place of Death, and Mortality Rates for Abdominal Aortic Aneurysm–Related Deaths Among U.S. Adults Aged ≥ 65 Years, United States, 1999–2024; Table S2: Annual percent change (APC) and Average annual percent change (AAPC) of Abdominal Aortic Aneurysm-related mortality stratified by overall, sex, Census, Race, Urbanization, and Age group among adults aged ≥ 65 years in the United States, 1999–2024.

## References

[1] Chaikof E.L., Dalman R.L., Eskandari M.K., Jackson B.M., Lee W.A., Mansour M.A., Mastracci T.M., Mell M., Murad M.H., Nguyen L.L. (2018). The Society for Vascular Surgery practice guidelines on the care of patients with an abdominal aortic aneurysm. J. Vasc. Surg..

[2] Reimerink J.J., Van Der Laan M.J., Koelemay M.J., Balm R., Legemate D.A. (2013). Systematic review and meta-analysis of population-based mortality from ruptured abdominal aortic aneurysm. Br. J. Surg..

[3] Hoornweg L.L., Storm-Versloot M.N., Ubbink D.T., Koelemay M.J.W., Legemate D.A., Balm R. (2008). Meta Analysis on Mortality of Ruptured Abdominal Aortic Aneurysms. Eur. J. Vasc. Endovasc. Surg..

[4] Nemade H., Mehrkens D., Lottermoser H.S., Yilmaz Z.E., Mader J., Schelemei P., Picard F.R., Geißen S., Schwab G.F., Hoyer F.F. (2026). Loss of MLKL impairs abdominal aortic aneurysm development by attenuating smooth muscle cell necroptosis. Cell Death Dis..

[5] Kent K.C. (2014). Abdominal Aortic Aneurysms. N. Engl. J. Med..

[6] Kuivaniemi H., Ryer E.J., Elmore J.R., Tromp G. (2015). Understanding the pathogenesis of abdominal aortic aneurysms. Expert Rev. Cardiovasc. Ther..

[7] Lo R.C., Schermerhorn M.L. (2016). Abdominal aortic aneurysms in women. J. Vasc. Surg..

[8] Marzano A., Flora F., Jabbour J., Martinelli O., Cuozzo S. (2026). Sex Differences in Outcomes After Endovascular Abdominal Aortic Aneurysm Repair: A Systematic Review and Narrative Synthesis. Rev. Cardiovasc. Med..

[9] Wang Z., You Y., Yin Z., Bao Q., Lei S., Yu J., Xie C., Ye F., Xie X. (2022). Burden of Aortic Aneurysm and Its Attributable Risk Factors from 1990 to 2019: An Analysis of the Global Burden of Disease Study 2019. Front. Cardiovasc. Med..

[10] Goyal A., Saeed H., Shahnoor S., Arshad M.K., Wasay A., Abdullah, Daoud M., Sohail A.H. (2024). Mortality trends, sex, and racial disparities in older adults due to abdominal aortic aneurysm: A nationwide cross-sectional analysis. Int. J. Surg..

[11] Ahmad M.S., Imran S., Rab A.A., Shahzad W., Nawaz I., Hannan A., Shweliya M.A. (2026). Mortality Trends for Aortic Aneurysm Rupture in the United States, 1999–2020: A Population-Based Observational Study Using CDC WONDER. Ann. Vasc. Surg..

[12] Sidloff D., Stather P., Dattani N., Bown M., Thompson J., Sayers R., Choke E. (2014). Aneurysm Global Epidemiology Study: Public Health Measures Can Further Reduce Abdominal Aortic Aneurysm Mortality. Circulation.

[13] Anjum A., Von Allmen R., Greenhalgh R., Powell J.T. (2012). Explaining the decrease in mortality from abdominal aortic aneurysm rupture. Br. J. Surg..

[14] Lederle F.A. (2011). The Rise and Fall of Abdominal Aortic Aneurysm. Circulation.

[15] Forsdahl S.H., Singh K., Solberg S., Jacobsen B.K. (2009). Risk Factors for Abdominal Aortic Aneurysms: A 7-Year Prospective Study: The Tromsø Study, 1994–2001. Circulation.

[16] Golledge J., Muller J., Daugherty A., Norman P. (2006). Abdominal Aortic Aneurysm: Pathogenesis and Implications for Management. Arterioscler. Thromb. Vasc. Biol..

[17] Sweeting M.J., Thompson S.G., Brown L.C., Powell J.T. (2012). Meta-analysis of individual patient data to examine factors affecting growth and rupture of small abdominal aortic aneurysms. Br. J. Surg..

[18] Scott R. (2002). The Multicentre Aneurysm Screening Study (MASS) into the effect of abdominal aortic aneurysm screening on mortality in men: A randomised controlled trial. Lancet.

[19] Harris P.L., Vallabhaneni S.R., Desgranges P., Becquemin J.P., van Marrewijk C., Laheij R.J. (2000). Incidence and risk factors of late rupture, conversion, and death after endovascular repair of infrarenal aortic aneurysms: The EUROSTAR experience. European Collaborators on Stent/graft Techniques for Aortic Aneurysm Repair. J. Vasc. Surg..

[20] EVAR trial participants (2005). Endovascular aneurysm repair versus open repair in patients with abdominal aortic aneurysm (EVAR trial 1): Randomised controlled trial. Lancet.

[21] Schermerhorn M.L., Buck D.B., O’Malley A.J., Curran T., McCallum J.C., Darling J., Landon B.E. (2015). Long-Term Outcomes of Abdominal Aortic Aneurysm in the Medicare Population. N. Engl. J. Med..

[22] WHO Director-General’s Opening Remarks at the Media Briefing on COVID-19—11 March 2020. https://www.who.int/news-room/speeches/item/who-director-general-s-opening-remarks-at-the-media-briefing-on-covid-19---11-march-2020.

[23] Msemburi W., Karlinsky A., Knutson V., Aleshin-Guendel S., Chatterji S., Wakefield J. (2023). The WHO estimates of excess mortality associated with the COVID-19 pandemic. Nature.

[24] Xu X., Shi Z., Zhou L., Lin J., Atlantis E., Chen X., Hussain A., Wang Y., Wang Y. (2024). Impact of COVID-19 on risks and deaths of non-communicable diseases in the Western Pacific region. Lancet Reg. Health West. Pac..

[25] Gupta R., Mouawad N.J., Yi J.A. (2021). The impact of the COVID-19 pandemic on vascular surgery: Health care systems, economic, and clinical implications. Semin. Vasc. Surg..

[26] Lange S.J., Ritchey M.D., Goodman A.B., Dias T., Twentyman E., Fuld J., Schieve L.A., Imperatore G., Benoit S.R., Kite-Powell A. (2020). Potential Indirect Effects of the COVID-19 Pandemic on Use of Emergency Departments for Acute Life-Threatening Conditions—United States, January–May 2020. MMWR Morb. Mortal. Wkly. Rep..

[27] Ng J.J., Ho P., Dharmaraj R.B., Wong J.C.L., Choong A.M.T.L. (2020). The global impact of COVID-19 on vascular surgical services. J. Vasc. Surg..

[28] Cao Z., Gao J., Wu J., Zheng Y. (2024). The Impact of COVID-19 Infection on Abdominal Aortic Aneurysms: Mechanisms and Clinical Implications. Cardiovasc. Ther..

[29] Moynihan R., Sanders S., Michaleff Z.A., Scott A.M., Clark J., To E.J., Jones M., Kitchener E., Fox M., Johansson M. (2021). Impact of COVID-19 pandemic on utilisation of healthcare services: A systematic review. BMJ Open.

[30] Czeisler M.É., Marynak K., Clarke K.E.N., Salah Z., Shakya I., Thierry J.M., Ali N., McMillan H., Wiley J.F., Weaver M.D. (2020). Delay or Avoidance of Medical Care Because of COVID-19–Related Concerns—United States, June 2020. MMWR Morb. Mortal. Wkly. Rep..

[31] COVIDSurg Collaborative (2020). Elective surgery cancellations due to the COVID-19 pandemic: Global predictive modelling to inform surgical recovery plans: Elective surgery during the SARS-CoV-2 pandemic. Br. J. Surg..

[32] CDC WONDER. https://wonder.cdc.gov/.

[33] Von Elm E., Altman D.G., Egger M., Pocock S.J., Gøtzsche P.C., Vandenbroucke J.P. (2007). The Strengthening the Reporting of Observational Studies in Epidemiology (STROBE) Statement: Guidelines for Reporting Observational Studies. PLoS Med..

[34] Joinpoint Regression Program. https://surveillance.cancer.gov/joinpoint/index.html.

[35] Norman P.E., Curci J.A. (2013). Understanding the effects of tobacco smoke on the pathogenesis of aortic aneurysm. Arterioscler. Thromb. Vasc. Biol..

[36] Isselbacher E.M. (2005). Thoracic and Abdominal Aortic Aneurysms. Circulation.

[37] Singh K., Bønaa K.H., Jacobsen B.K., Bjørk L., Solberg S. (2001). Prevalence of and risk factors for abdominal aortic aneurysms in a population-based study: The Tromso Study. Am. J. Epidemiol..

[38] Hannawa K.K., Eliason J.L., Upchurch G.R. (2009). Gender Differences in Abdominal Aortic Aneurysms. Vascular.

[39] Malayala S.V., Raza A., Vanaparthy R. (2020). Gender-Based Differences in Abdominal Aortic Aneurysm Rupture: A Retrospective Study. J. Clin. Med. Res..

[40] Boese A.C., Chang L., Yin K.J., Chen Y.E., Lee J.P., Hamblin M.H. (2018). Sex differences in abdominal aortic aneurysms. Am. J. Physiol.-Heart Circ. Physiol..

[41] Appah-Sampong A., Marcaccio C., Li S., Song Y., Hussain M.A., Yeh R., Schermerhorn M.L., Secemsky E.A. (2025). Racial Disparities in Long-Term Outcomes After Endovascular Aortic Aneurysm Repair in Black and White Medicare Beneficiaries. Circulation.

[42] Kent K.C., Zwolak R.M., Egorova N.N., Riles T.S., Manganaro A., Moskowitz A.J., Gelijns A.C., Greco G. (2010). Analysis of risk factors for abdominal aortic aneurysm in a cohort of more than 3 million individuals. J. Vasc. Surg..

[43] Barshes N.R., Bidare D., Kougias P., Mills J.L., LeMaire S.A. (2022). Racial and ethnic disparities in abdominal aortic aneurysm evaluation and treatment rates in Texas. J. Vasc. Surg..

[44] Kim J., Chung S.W., Kim J., Bae M., Lee C.W., Huh U. (2025). Analysis of Mortality-Related Factors in Patients Aged >80 Years Treated for Abdominal Aortic Aneurysms. J. Chest Surg..

[45] Altobelli E., Rapacchietta L., Profeta V.F., Fagnano R. (2018). Risk Factors for Abdominal Aortic Aneurysm in Population-Based Studies: A Systematic Review and Meta-Analysis. Int. J. Environ. Res. Public Health.

[46] Zettervall S.L., Soden P.A., Buck D.B., Cronenwett J.L., Goodney P.P., Eslami M.H., Lee J.T., Schermerhorn M.L. (2017). Significant regional variation exists in morbidity and mortality after repair of abdominal aortic aneurysm. J. Vasc. Surg..

[47] Lederle F.A., Johnson G.R., Wilson S.E., Chute E.P., Littooy F.N., Bandyk D., Krupski W.C., Barone G.W., Acher C.W., Ballard D.J. (1997). Prevalence and Associations of Abdominal Aortic Aneurysm Detected through Screening. Ann. Intern. Med..

