# Supplementary material for: Temporal Trends and Demographic Disparities in Abdominal Aortic Aneurysm Mortality Among U.S. Adults Aged ≥ 65 Years, 1999–2024: A Nationwide Population-Based Analysis of CDC WONDER Data
Source: J Clin Med. 2026 Jul 1;15(13):5130. doi: 10.3390/jcm15135130 (PMC13362732; doi:10.3390/jcm15135130)
Supplement: Supplementary file 1 [file jcm-15-05130-s001.zip › Supplementary Figures.pdf]

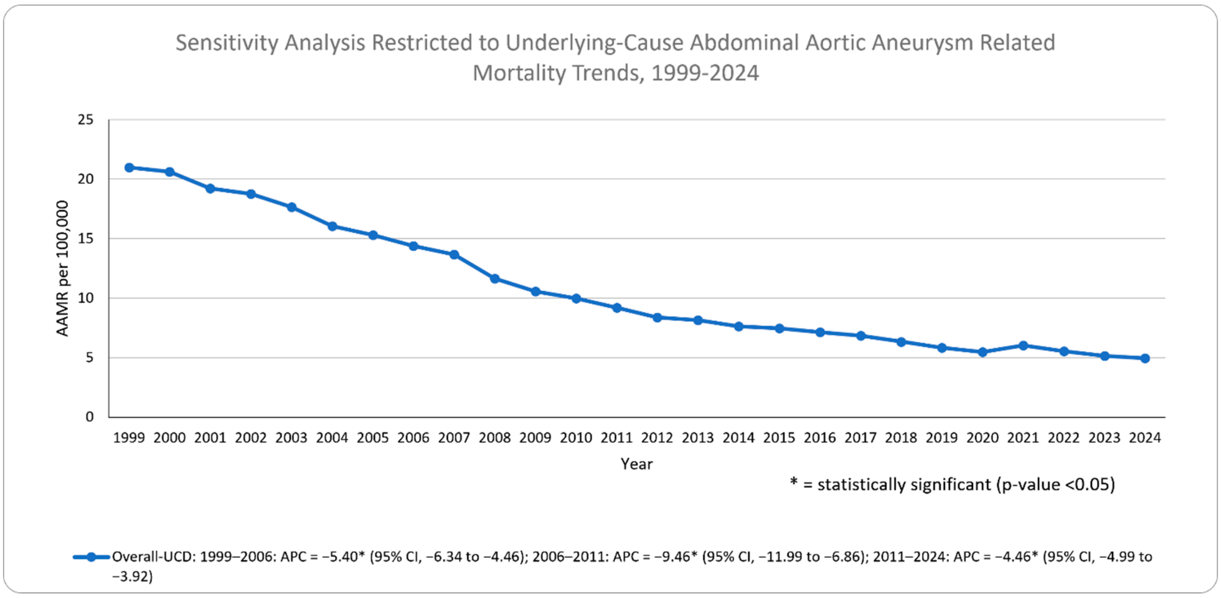

Figure S1: Sensitivity Analysis

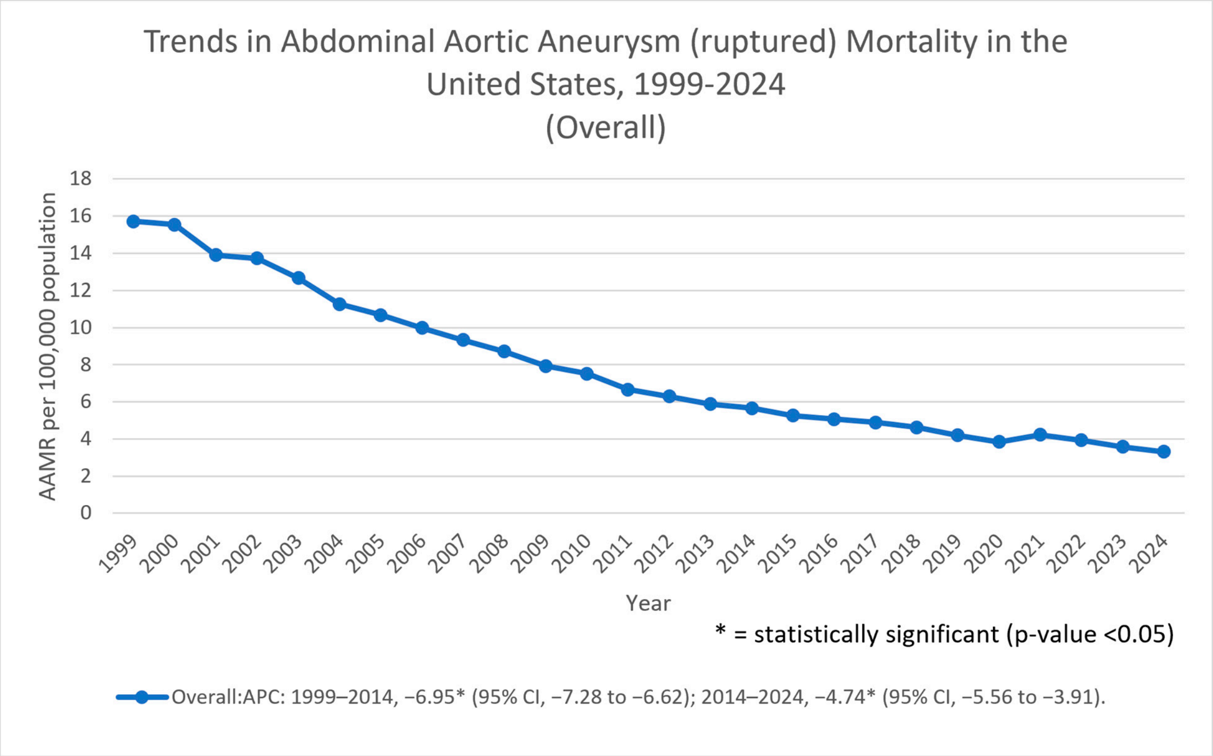

Figure S2: Trends in Abdominal Aortic Aneurysm (ruptured) Mortality in the United States, 1999-2024

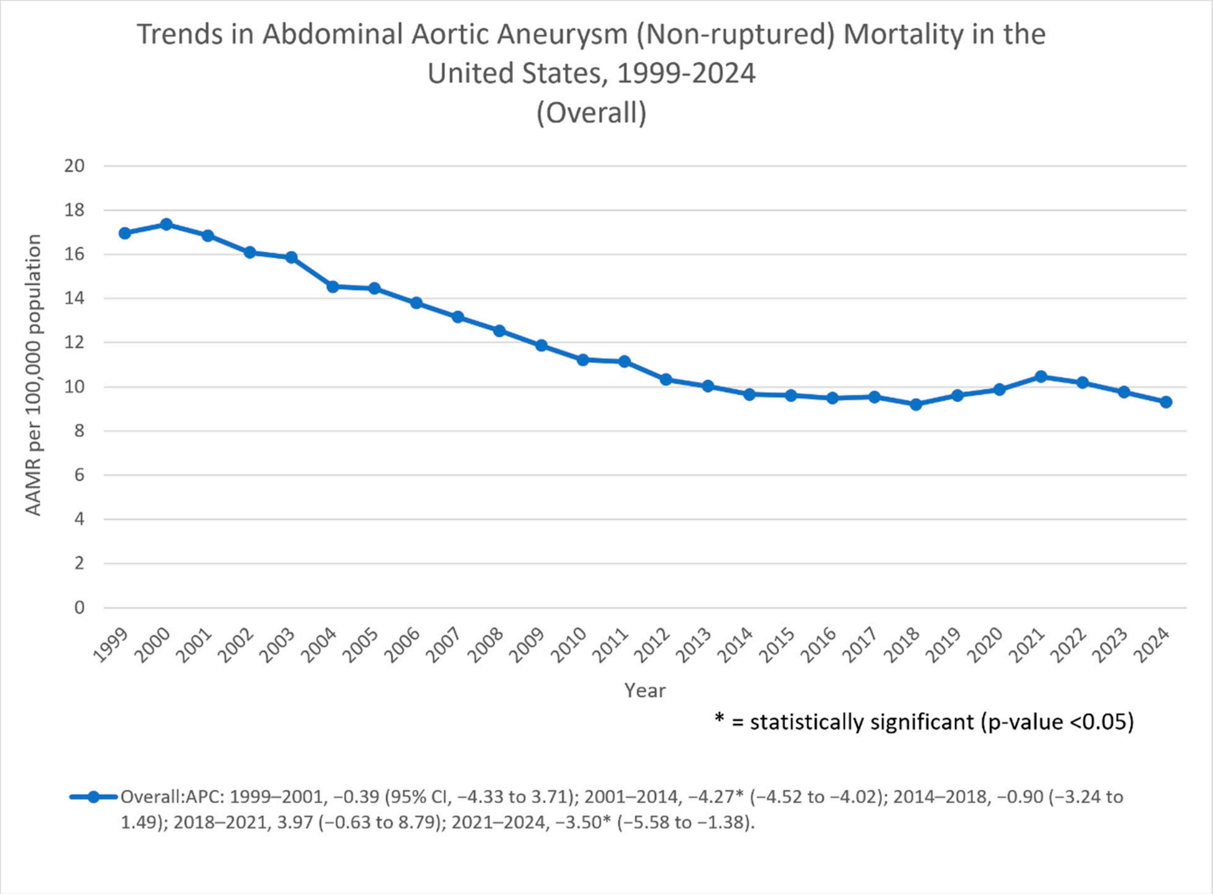

Figure S3: Trends in Abdominal Aortic Aneurysm (Non-ruptured) Mortality in the United States, 1999-2024
