# Supplementary material for: Temporal Trends and Demographic Disparities in Abdominal Aortic Aneurysm Mortality Among U.S. Adults Aged ≥ 65 Years, 1999–2024: A Nationwide Population-Based Analysis of CDC WONDER Data
Source: J Clin Med. 2026 Jul 1;15(13):5130. doi: 10.3390/jcm15135130 (PMC13362732; doi:10.3390/jcm15135130)
Supplement: Supplementary file 1 [file jcm-15-05130-s001.zip › Supplemntary Table S1.pdf]

Supplementary Table S1: Demographic Characteristics, Place of Death, and Mortality Rates for Abdominal Aortic Aneurysm–Related Deaths Among U.S. Adults Aged ≥65 Years, United States, 1999–2024

Characteristic	Deaths (N)	%	AAMR 1999	AAMR 2024
Overall	208,476	100.0	32.61	12.17
**Sex**				
Female	76,171	36.5	18.48	7.02
Male	132,305	63.5	55.61	19.17
**Census Region**				
Northeast	42,131	20.2	34.17	11.52
Midwest	55,259	26.5	37.69	14.50
South	69,082	33.1	29.71	11.44
West	42,004	20.1	29.81	11.89
**Race/Ethnicity**				
American Indian or Alaska Native	744	0.36	26.11	9.91
Asian or Pacific Islander	3,901	1.87	19.07	6.40
Black or African American	11,126	5.34	19.85	9.06
White	186,135	89.28	35.07	13.86
Hispanic	6,007	2.88	13.84	5.67
**Urbanization (1999–2020) [a]**				
Metropolitan	140,609	67.4	31.55	12.43 (2020)
Non-Metropolitan	39,428	18.9	37.24	17.24 (2020)
**Age Group (CMR) [b]**				
65–74 years	49,158	23.6	16.74	4.82
75–84 years	86,075	41.3	41.78	14.22
85+ years	73,243	35.1	73.81	37.65
**Place of Death [c]**				
Medical Facility- Inpatient	101806	48.8	—	—
Medical Facility - Outpatient or ER	28151	13.5	—	—
Medical Facility - Dead on Arrival	1213	0.58	—	—
Medical Facility - Status unknown	248	0.11	—	—
Decedent Home	42,844	20.6		
Nursing Home	22,838	11.0	—	—

Characteristic	Deaths (N)	%	AAMR 1999	AAMR 2024
Hospice	5,553	2.7	—	—
Other	5,351	2.6	—	—
Unknown	471	0.2	—	—

[a] Urbanization data was available till 2020.
[b] Crude Mortality Rate is used for analysis instead of Age-Adjusted Mortality Rates for Age groups.
[c] AAMR is not applicable for Place of Death.
