# Supplementary material for: Temporal Trends and Demographic Disparities in Abdominal Aortic Aneurysm Mortality Among U.S. Adults Aged ≥ 65 Years, 1999–2024: A Nationwide Population-Based Analysis of CDC WONDER Data
Source: J Clin Med. 2026 Jul 1;15(13):5130. doi: 10.3390/jcm15135130 (PMC13362732; doi:10.3390/jcm15135130)
Supplement: Supplementary file 1 [file jcm-15-05130-s001.zip › Supplmentary Table S2 APC and AAPC.pdf]

Supplementary Table S2: Annual percent change (APC) and Average annual percent change (AAPC) of Abdominal Aortic Aneurysm-related mortality stratified by overall, sex, Census, Race, Urbanization, and Age group among adults aged ≥65years in the United States, 1999–2024.

Characteristic	Trend Period	APC* (95% CI)	AAPC* (95% CI)	p-value
Entire Cohort	1999–2002	-4.04* (-5.59 to -2.47)	-3.86* (-4.42 to -3.30)	< 0.000001
	2002–2013	-5.69* (-5.97 to -5.42)		
	2013–2018	-2.89* (-4.16 to -1.60)		
	2018–2021	2.12 (-1.98 to 6.39)		
	2021–2024	-4.32* (-6.26 to -2.35)		
**Sex**				
Female	1999–2007	-3.88* (-4.35 to -3.41)	-3.82* (-4.60 to -3.03)	< 0.000001
	2007–2013	-5.81* (-6.87 to -4.74)		
	2013–2019	-2.88* (-4.06 to -1.68)		
	2019–2022	0.21 (-5.17 to 5.91)		
	2022–2024	-6.21* (-11.37 to -0.75)		
Male	1999–2002	-4.83* (-6.84 to -2.78)	-4.25* (-4.90 to -3.60)	< 0.000001
	2002–2013	-6.41* (-6.77 to -6.04)		
	2013–2018	-2.64* (-4.31 to -0.94)		
	2018–2022	1.97 (-0.66 to 4.67)		
	2022–2024	-7.36* (-12.13 to -2.33)		
**US Census Region**				
Northeast	1999–2016	-5.69* (-5.93 to -5.45)	-4.31* (-5.00 to -3.62)	< 0.000001
	2016–2021	0.76 (-1.97 to 3.56)		
	2021–2024	-4.64* (-8.66 to -0.44)		
Midwest	1999–2006	-4.49* (-4.94 to -4.04)	-3.80* (-4.39 to -3.20)	< 0.000001
	2006–2012	-6.48* (-7.34 to -5.61)		
	2012–2018	-2.48* (-3.46 to -1.49)		
	2018–2021	2.58 (-1.83 to 7.20)		
	2021–2024	-5.49* (-7.57 to -3.37)		
South	1999–2001	-2.47 (-7.28 to 2.58)	-3.79* (-4.71 to -2.86)	< 0.000001
	2001–2014	-5.82* (-6.14 to -5.49)		
	2014–2019	-1.57 (-3.54 to 0.45)		

Characteristic	Trend Period	APC* (95% CI)	AAPC* (95% CI)	p-value
	2019–2022	2.52 (-3.62 to 9.05)		
	2022–2024	-6.41* (-11.97 to -0.50)		
West	1999–2014	-4.83* (-5.25 to -4.42)	-3.58* (-3.97 to -3.19)	< 0.000001
	2014–2024	-1.66* (-2.50 to -0.82)		
**Race/Ethnicity**				
American Indian or Alaska Native	1999–2017	-5.10* (-6.28 to -3.91)	-3.15* (-4.59 to -1.70)	0.000026
	2017–2024	2.04 (-2.61 to 6.92)		
Asian or Pacific Islander	1999–2018	-5.06* (-5.64 to -4.47)	-4.01* (-4.79 to -3.23)	< 0.000001
	2018–2024	-0.62 (-3.58 to 2.44)		
Black or African American	1999–2014	-4.95* (-5.55 to -4.36)	-3.29* (-3.84 to -2.74)	< 0.000001
	2014–2024	-0.74 (-1.91 to 0.44)		
White	1999–2002	-3.92* (-5.42 to -2.40)	-3.64* (-4.19 to -3.08)	< 0.000001
	2002–2013	-5.54* (-5.81 to -5.27)		
	2013–2018	-2.55* (-3.80 to -1.28)		
	2018–2021	2.39 (-1.66 to 6.61)		
	2021–2024	-3.95* (-5.86 to -2.01)		
Hispanic or Latino	1999–2014	-5.25* (-6.30 to -4.18)	-3.70* (-4.60 to -2.80)	< 0.000001
	2014–2024	-1.34 (-3.12 to 0.47)		
**Urbanization (1999–2020 only)**				
Metropolitan	1999–2014	-5.53* (-5.71 to -5.35)	-4.54* (-4.79 to -4.29)	< 0.000001
	2014–2020	-2.03* (-2.86 to -1.18)		
Non-Metropolitan	1999–2007	-4.24* (-4.90 to -3.57)	-3.74* (-4.31 to -3.17)	< 0.000001
	2007–2013	-6.29* (-7.84 to -4.71)		
	2013–2020	-0.91 (-1.95 to 0.13)		
**Age Group**				
65–74 Years	1999–2013	-7.71* (-8.10 to -7.31)	-4.84* (-5.19 to -4.48)	< 0.000001
	2013–2024	-1.06* (-1.75 to -0.36)		
75–84 Years	1999–2002	-3.68* (-6.45 to -0.83)	-4.27* (-5.03 to -3.50)	< 0.000001
	2002–2016	-5.79* (-6.15 to -5.43)		
	2016–2022	-0.17 (-1.99 to 1.67)		

Characteristic	Trend Period	APC* (95% CI)	AAPC* (95% CI)	p-value
	2022–2024	-6.40 (-13.51 to 1.30)		
85+ Years	1999–2005	-2.75* (-3.47 to -2.02)	-2.66* (-3.35 to -1.97)	< 0.000001
	2005–2014	-4.08* (-4.55 to -3.61)		
	2014–2019	-1.51* (-2.97 to -0.03)		
	2019–2022	2.46 (-2.25 to 7.40)		
	2022–2024	-6.22* (-10.64 to -1.57)		

APC = Annual Percent Change; AAPC = Average Annual Percent Change; CI = Confidence Interval; * $p < 0.05$ (statistically significant trend). Urbanization data available through 2020 only.
